# Secondary analysis of an RCT on Emergency Department-Initiated Tobacco Control: Repeatedly assessed point-prevalence abstinence up to 12 months and extension of results through a 10-year follow-up

**DOI:** 10.18332/tid/105579

**Published:** 2019-04-05

**Authors:** Edith Weiss-Gerlach, William J. McCarthy, Jürgen Wellmann, Marie Graunke, Claudia Spies, Bruno Neuner

**Affiliations:** 1 Department of Anesthesiology and Intensive Care Medicine, Charité– Universitätsmedizin Berlin, Berlin, Germany; 2 Center for Cancer Prevention and Control Research, UCLA Fielding School of Public Health, Los Angeles, United States; 3 Institute of Epidemiology and Social Medicine, University of Münster, Münster, Germany

**Keywords:** tobacco use cessation, emergency service, hospital, long-term follow-up

## Abstract

**INTRODUCTION:**

Emergency departments (EDs) are opportune places for tobacco control interventions. The ‘Tobacco Control in an Urban Emergency Department’ (TED) study, ISRCTN41527831, originally evaluated the effect of motivational interviewing on-site plus up to four booster telephone calls on 12-month abstinence. This study’s aim was to evaluate the effect of the intervention on 7-day point-prevalence abstinence at 10 years follow-up (primary outcome) as well as on repeated point-prevalence abstinence at 1, 3, 6, 12 months and at 10 years (continual smoking abstinence, secondary outcome).

**METHODS:**

At the 10 years follow-up and after informed consent, study participants responded to a mailed questionnaire. The primary outcome was analyzed in observed-only and in all-cases analyses. The secondary outcomes were analyzed using a multiple adjusted GLMM for binary outcomes.

**RESULTS:**

Out of 1012 TED-study participants, 986 (97.4%) were alive and 231 (23.4% of 986) responded to the follow-up at 10 years. For observed-only and all-cases analyses, the effect of the baseline intervention on 7-day point-prevalence abstinence at the 10 years follow-up was statistically non-significant. However, when taking into account all repeated measures, the intervention significantly influenced continual abstinence with odds ratio 1.32 (95% CI: 1.01–1.73; p=0.042). Baseline motivation, perceived self-efficacy to stop smoking, and nicotine dependency were independently associated with long-term continual smoking abstinence (all p<0.05).

**CONCLUSIONS:**

A conventional analysis failed to confirm a significant effect of the ED-initiated tobacco control intervention on the point-prevalence abstinence at 10 years. Results from a more integrative analysis nonetheless indicated an enduring intervention effect on continual abstinence among smokers first encountered in the emergency department setting 10 years earlier.

## ABBREVIATIONS

GLMM: generalized linear mixed model
TED: Tobacco Control in an Urban Emergency Department
ED: emergency department
RCT: randomized controlled trial
Laocoon: Long-Term-Follow-up of Tobacco Control in an Urban Emergency Department

## INTRODUCTION

Emergency departments (EDs) are opportune settings for promoting healthier lifestyle behaviors and preventing disease, including cigarette smoking^1,2^. A 2006 recommendation of the American College of Emergency Physicians advocated routine screening of ED patients’ smoking status and to advise ED smokers to quit^3^. The recommendation introduced the term ‘Emergency Department-Initiated Tobacco Control’ for such procedures, typically a combination of on-site motivational interviewing/counseling, followed by either booster interventions or referral to other institutions providing smoking cessation counseling^3^. Since 2000, several randomized controlled trials (RCTs) evaluated the efficacy of such screening and brief intervention efforts, mostly a combination of an initial motivational counselling session and either booster intervention sessions or referral to outpatient services, including tobacco quitlines. A recent systematic review and meta-analysis identified 11 RCTs and a significant effect of ED-initiated tobacco control on 7-day point-prevalence tobacco abstinence at 1 and 3 months follow-up^4^. Additionally, a statistically significant benefit from exposure to the ED-initiated tobacco control services was observed, using repeated 7-day point-prevalence tobacco abstinence measures (continual tobacco-use abstinence) up to 12 months post-intervention. Pooling the results of all available studies and across all follow-up time points, patients in the intervention group showed a relative risk (RR) of 1.40 (95% CI: 1.06–1.86; p=0.02), of being tobacco abstinent^4^. Thus, their chance of being at least momentarily abstinent increased by 40%. Continual tobacco-use abstinence may be regarded as an important precursor to continuous abstinence, the ultimate goal of tobacco cessation efforts for several reasons: 1) even transient periods of smoking abstinence may yield positive health impact, for example, on lung function^5^; 2) previous experience with temporary abstinence predisposes to further attempts to quit^6^; and 3) smokers typically need several quit attempts before achieving long-term abstinence^7^. The aim of the current study was therefore two-fold: First, a 10-year follow-up allowed us to evaluate the long-term outcome of an ED-initiated tobacco control intervention 10 years after the initial RCT was stopped^8^. Secondly, by evaluating continual abstinence instead of the 7-day point-prevalence tobacco abstinence at the 12 months follow-up, we re-evaluated the initial RCT on ED-initiated tobacco control^8^ and extended the analysis of continual abstinence status to follow-up over 10 years. Finally, the multivariable analysis of these parameters allowed the identification of baseline parameters independently associated with long-term continual abstinence.

## METHODS

### Data sources

The parent study was the randomized, controlled trial entitled ‘Tobacco Control in an Urban Emergency Department’ (TED), ISRCTN41527831^8^. This single-center study was conducted in 2005/2006 at one campus of the Charité–Universitaetsmedizin Berlin, Germany, with the last follow-up taking place in January 2008. The emergency department at this campus has two subunits, one for surgical and one for internal medicine patients. The study was conducted in both units. Briefly, 1012 out of 1044 randomized urban emergency department patients had a complete baseline assessment and received either motivational interviewing on-site in combination with up to four booster phone calls, or usual care. Usual care patients received a brief leaflet with some general information on smoking cessation but no counselling on-site and no booster phone calls. Follow-up assessment started one month after the motivational interviewing on-site and was repeated at 3, 6, and 12 months follow-up. The predefined outcome was the 7-day point-prevalence abstinence at the 12 months follow-up, showing an adjusted intervention effect versus usual care (OR=1.31; 95% CI: 0.91–1.89; p=0.15)^8^.

### Ten-year follow-up

The ‘Long-Term-Follow-up of Tobacco Control in an Urban Emergency Department’ (Laocoon) study received ethical committee approval (EA1/238/15) in June 2015. Out of 1012 TED-study participants with complete baseline questionnaire, 122 (12.1%) were not reachable at the follow-up at 12 months. Thus, initially 890 study participants received a mailed ‘study package’, consisting of the study information, the consent form and the study questionnaire together with a business reply envelope. Successively undeliverable mailing addresses, together with the 122 addresses known to be incorrect were transferred to Address Research^®^, a service provider from the German postal service (Deutsche Post AG). This service provider has access to official registration office data and provides information on national addresses, information on tenancy changeover abroad (with no further information on target country or a specific address) and, in case of death, information on the mortality status. Newly retrieved addresses were continuously added into the contact algorithm. Study participants of the TED study, who were alive at the time of the Laocoon study and who had a known current address received the initial study package and two postal reminders. A last attempt was made to reach potential eligible participants by sending a 2-page questionnaire, evaluating the main study outcome.

### Study measures

All study measures were evaluated by self-administered postal questionnaire. The predefined primary outcome was the 7-day point-prevalence abstinence at the 10 years follow-up. This outcome was assessed using two questions: ‘Did you smoke during the past seven days?’. Those, who answered ‘daily’ or ‘on xx days’, were asked: ‘During the days on which you smoked, how many cigarettes did you usually smoke per day?’. Further smoking-status related questions concerned the degree of nicotine dependency, measured with the Fagerström Test for Nicotine Dependency (FTND)^9^, motivation to stop smoking, assessed with a single question ‘When do you wish to stop smoking?’ with three possible answers: ‘Not within the next 6 months’=unmotivated smokers, ‘Within the next 6 months but not within the next 4 weeks’=ambivalent smokers and ‘Within the next 4 weeks’=motivated smokers^10,11^. Further questions evaluated illicit drug use^8^, alcohol consumption^12^, socioeconomic status^8^ and the participant’s current medical status. The 2-page questionnaire evaluated the main study outcome, the heaviness of smoking index (HSI^13^, a short-form of the FTND^9^), motivation to stop smoking^10,11^ and current medical status.

A secondary study outcome was repeated point-prevalence abstinence through 10 years of follow-up (i.e. 7-day point-prevalence tobacco abstinence at 1, 3, 6, 12 months and at 10 years, continual abstinence). Covariates for adjustments of the multivariable models (see Statistical Analysis section) were chosen based on their association (p<0.1)^14,15^ with participant non-response (Table 1) or because the scientific literature had identified them as recognized predictors of smoking counseling outcomes, these were: emergency department subunit (surgical or internal medicine), age, level of nicotine dependency (Fagerström Test), partnership status, educational level (university-entrance diploma), having a family doctor, gender, motivation to stop smoking (10-point scale from 1 to 10 points according to Miller and Rollnick^16^) and perceived self-efficacy to stop smoking (10-point scale from 1 to 10 points according to Miller and Rollnick^17^). The lower pole of the two scales referred to no motivation/no self-efficacy to stop smoking while the upper pole indicated very high motivation/ self-efficacy to stop smoking.

**Table 1:** Predictors of repeated point prevalence abstinence up to 12 months and up to 10 years after emergency department-initiated tobacco control, results of GLMM analyses (N=1011 )^a^

*Baseline parameter at study entry (TED study)*	*Model 1: Follow-up through 12 months only*			*Model 2: Follow-up through 10 years*		
		*OR ( 95% CI)*	*p*		*OR ( 95% CI)*	*p*
Intervention group vs control group	unadjusted^b^	1.32 (0.99–1.77)	0.062	unadjusted^b^	1.32 (1.02–1.72)	0.035
Intervention group vs control group	adjusted^c^	1.28 (0.95–1.74)	0.103	adjusted^c^	1.32 (1.01–1.73)	0.042
Internal medicine subunit vs surgical subunit		1.42 (1.04–1.93)	0.027		1.32 (1.01–1.74)	0.044
Age (years), per additional year		1.01 (0.99–1.02)	0.40		1.01 (0.99–1.02)	0.36
Male vs Female gender		0.97 (0.71–1.32)	0.84		0.93 (0.71–1.23)	0.63
Fagerström test, per additional point		0.88 (0.82–0.95)	0.001		0.89 (0.83–0.94)	< 0.001
Motivation to stop smoking^d^, per additional point		1.20 (1.13–1.28)	< 0.001		1.19 (1.13–1.25)	< 0.001
Perceived self-efficacy to stop smoking^e^, per additional point		1.18 (1.11–1.26)	< 0.001		1.15 (1.09–1.22)	< 0.001
**Partnership**						
Non-smoking partner vs no partnership		1.03 (0.71–1.50)	0.87		1.17 (0.84–1.62)	0.36
Smoking partner vs no partnership		0.73 (0.51–1.05)	0.087		0.74 (0.54–1.03)	0.071
University-entrance diploma vs none		1.17 (0.86–1.60)	0.32		1.21 (0.92–1.60)	0.17
Family doctor vs none		1.11 (0.79–1.57)	0.55		1.16 (0.85–1.58)	0.34

a 1012 participants of the TED study with complete baseline questionnaire data minus one study participant who withdrew his initial participation consent.

b With time to follow-up as ordinal term and interaction intervention group×time linear (p>0.9).

c Plus adjustments.

d 10-point scale from 1 to 10 points with higher points indicating higher motivation to stop smoking^16^.

e 10-point from 1 to 10 points with higher points indicating higher perceived self-efficacy to stop smoking^17^. TED: Tobacco Control in an Urban Emergency Department^8^. GLMM: generalized linear mixed model. OR: odds ratio. CI: confidence interval.

### Statistical analysis

The patients’ flow through the follow-up is graphically displayed by a lasagna-plot^18^, a way of showing for all study participants the individual course of smoking/non-smoking and study attrition transitions over all follow-up times.

Binary and ordinal variables are depicted as absolute and relative numbers. For variables with normal distributions, the measures of central tendency used are means with standard deviations, while for non-normally distributed variables, medians and their ranges are used. Differences between two independent groups were evaluated in normally distributed variables and non-normally distributed variables using Student’s t-test and Mann-Whitney-U-Test, respectively. For binary data such as the primary outcome of the Laocoon study (current smoking/non-smoking status), comparisons between groups were done using the chi-squared test.

The secondary study outcome, the effect of the ED-initiated tobacco control intervention on continual abstinence assessed repeatedly until the assessment at 12 months (totaling four follow-up times) and assessment at 10 years (thus totaling five follow-up times), was analyzed by logistic regression for repeated, correlated binary outcomes (generalized linear mixed models, GLMMs). The GLMM accounts for heterogeneity in the effect of the initial tobacco control intervention between subjects as well as for heterogeneity in the effect over time. A Markov-type Ante-Dependence covariance structure^19,20^ was employed for the marginal covariance matrix of each participant, which entails four and five different variance parameters, until 12 months (fourth follow-up) and until 10 years (fifth follow-up), respectively. This approach avoids repeated single tests of repeated measurements without taking the correlation with the preceding measurement and the preceding test statistics into account^19^. It is reasonable to assume a decaying correlation structure between repeated pairs of measurements over successive assessment intervals and thus a fading-off of the initial tobacco control intervention over time. Therefore, three (12-month follow-up, Model 1 in Table 1) and four (10-year follow-up, Model 2 in Table 1) additional covariances were included to capture the variability attributable to the transition from one follow-up examination to the next and to capture the varying time intervals between consecutive follow-up assessments (between one month and nine years). These correlations may be estimated from the data and are shown in Supplementary Table 1. The Ante-Dependence covariance structure is also robust in settings with dropouts unrelated to the ‘treatment’^19^, i.e. the tobacco cessation counseling in this study (Table 2; eligibility at 10 years was not associated with randomization status). The randomly assigned treatment group at baseline, time of follow-up assessment (one, three, six and twelve months as well as 10 years after baseline), and their interaction term were entered into the model as categorical variables. The interaction term was meant to capture an eventual differential effect of time in both study arms. The interpretation of the main effect of the intervention is that of an overall effect of the treatment across all time-points. The model was formulated using ‘Proc glimmix’ in SAS and the Ante-Dependence covariance structure was defined using the random statement:

**Table 2:** Baseline characteristics at the time of inclusion in the TED study in Laocoon-study participants, Laocoon-study non-responder and patients with indeterminate postal address (N=986 )

*Baseline and follow-up measures (TED study)*	*Current determinate postal address*			*Indeterminate postal address*
	*Study participants N=231 (23.4%)*	*Non-responder N=454 (46.0%)*	*p*	*N=301 (30.5%)*
Intervention group, n (%)	119 (51.5)	214 (47.1)	0.28	160 (53.2)
Surgical subunit of the ED, n (%)	125 (54.1)	214 (47.1)	0.084	137 (45.5)
Non-smoker at 1 month follow-up (N=654) n (%)	17 (9.5)	21 (7.3)	0.39	19 (10.2)
Non-smoker at 3 months follow-up (N=621) n (%)	25 (14.0)	33 (11.7)	0.48	23 (14.3)
Non-smoker at 6 months follow-up (N=633) n (%)	35 (18.9)	49 (16.9)	0.57	27 (17.1)
Non-smoker at 12 months follow-up (N=671) n (%)	47 (23.6)	49 (15.9)	0.031	33 (20.1)
Age (years) mean ± SD, range	34.9 ± 12.0, (18–68)	31.9 ± 9.9, (18–73)	0.001	30.9 ± 9.3, (18–78)
Female gender, n (%)	103 (44.6)	174 (38.3)	0.14	111 (36.9)
Number of cigarettes smoked per day mean ± SD, range	15.0 ± 9.3, (1–40)	16.9 ± 9.5, (1–60)	0.013	17.0 ± 10.3, (1–60)
Fagerström test mean ± SD	2.9 ± 2.6	3.4 ± 2.5	0.019	3.6 ± 2.6
Motivation to stop smoking 10-point scale, mean ± SD	5.4 ± 2.7	5.7 ± 2.6	0.12	5.7 ± 2.5
**Motivation to stop smoking^a^, n (%)**				
unmotivated	135 (58.4)	253 (55.7)		151 (50.2)
ambivalent	66 (28.6)	143 (31.5)	0.66^c^	111 (36.9)
motivated	30 (13.0)	58 (12.8)^b^		39 (13.0)^b^
Perceived self-efficacy to stop smoking 10-point scale, mean ± SD	5.6 ± 2.9	5.5 ± 2.7	0.12	5.6 ± 2.6
Harmful alcohol consumption^d^, n (%)	81 (35.1)	150 (33.0)	0.60	107 (35.5)
Illicit drug use^e^, n (%)	127 (55.0)	250 (55.1)	0.98	192 (63.8)
**Partnership, n (%)**				
Non-smoking partner	73 (31.6)	123 (27.1)	0.089	78 (25.9)
Smoking partner	87 (37.7)	211 (46.5)		130 (43.2)
No partner	71 (30.7)	120 (26.4)		93 (30.9)
Living in a single household, n (%)	87 (37.7)	165 (36.3)	0.74	123 (40.9)
University-entrance diploma, n (%)	128 (55.4)	206 (45.4)	0.013	156 (51.8)
Net family income/month above median^f^ in 2006 (N=187) n (%)	72 (38.5)	138 (41.7)	0.48	80 (34.5)
Family doctor, n (%)	183 (79.2)	330 (72.7)	0.062	189 (62.8)

a ‘When do you wish to stop smoking?’ (‘Not within the next 6 months’=unmotivated smokers, ‘Within the next 6 months but not within the next 4 weeks’=ambivalent smokers and ‘Within the next 4 weeks’=motivated smokers)^10,11^.

b Does not sum up to 100% because of rounding error.

c Chi-squared test for trend;

d ≥5 points in the AUDIT-PC^12^.

e At least one single use within the last 12 months before the TED-study baseline: cannabis, ecstasy, other designer drugs, natural drugs (e.g. Peyote), cocaine, morphine, heroin or other opiates^8^.

f 1475€/month^8^. TED: Tobacco Control in an Urban Emergency Department^8^. SD: standard deviation. ED: emergency department. AUDIT-PC: Alcohol Use Disorders Identification Test–Piccinelli Consumption.

#### RANDOM time/subject = id type = ante(1) RESIDUAL

Although the baseline data were derived from a well-balanced RCT with no indication of residual confounding (see Table 1 of Nuener et al.^8^), an unadjusted model was considered to be a suboptimal solution to modeling effects over such a long time span. Adjusting for variables associated with loss to follow-up was meant to address potential biases caused by differential attrition.

A p-value of less than 0.05 was defined as statistically significant. All statistical analyses were run in SPSS (IBM SPSS Statistics for Windows, Version 25.0. Armonk, NY: IBM Corp) or in SAS University Edition^®^.

## RESULTS

**Figure 1:**
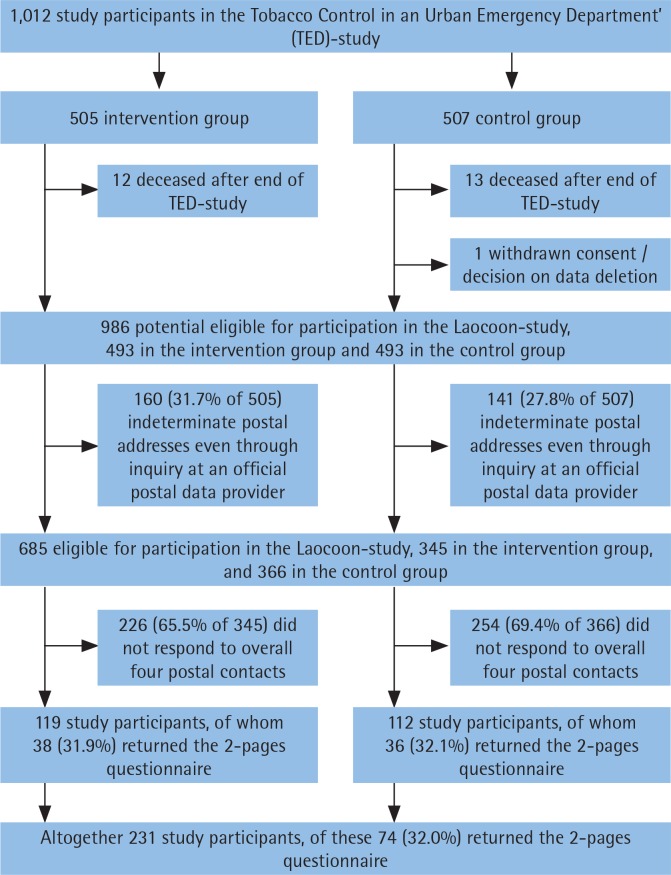
Flowchart of the study participation process (n=1012)

Out of 1012 TED-study participants with complete baseline questionnaire, 986 (97.3%) were potentially eligible for participation in the Laocoon study. Correct addresses were identified for 685 study participants (69.5%) and of these, 231 (33.7% of the 685) participated in the 10-year follow-up. Seventy-four study participants (32.0% of the 231) returned only the brief 2-page questionnaire. The details of the study participation/non-participation over time are shown in Figure 1.

**Figure 2:**
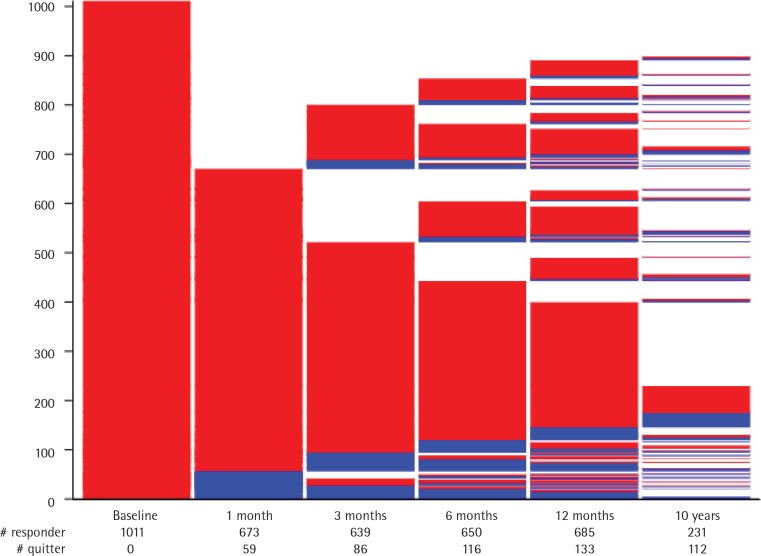
Lasagna-plot and marginal distribution table of smoking status and dropouts over all follow-up assessments for the TED and Laocoon studies (N=1011)

The lasagna-plot (Figure 2) shows the large proportion of those already lost to follow-up at 1 month (the white space above the second bar). However, with increasing duration of follow-ups and additional follow-up assessments, around two-thirds of those lost to follow-up at the assessment at 1 month ended up providing follow-up information about their smoking status at least one additional time. This held true even for those who were lost to follow-up between the 1 and 3 months follow-up assessments. Thus, over the course of the initial study period with a 12-month follow-up, more than 85% of study participants provided follow-up information at least once. On the other hand, many study participants who participated in the first follow-up assessment at one month did not provide further information or provided incomplete information in later follow-up assessments.

Regarding smoking status over consecutive assessments, only 7 out of 986 (0.7%) study participants were tobacco abstinent at all follow-up assessments and presumably stopped smoking permanently after the initial smoking cessation intervention. These 7 are represented on the lasagna-plot by the continual blue part of the five follow-up charts culminating in the sliver of blue at the bottom of the 6th assessment. Only 55 out of 986 (5.6%) study participants completed follow-up information indicating continual smoking at every assessment. These 55 are represented by the largest red ‘slice’ one-fifth from the bottom of the 5th follow-up assessment. Thus, nearly 94 per cent of study participants switched between smoking/non-smoking status and study participation/study attrition over the course of five assessments conducted over 10 years of follow-up.

The total number of non-smokers in both study groups and per follow-up interval is given in Supplementary Figure 1. The number of non-smokers in the intervention group exceeded the number of non-smokers in the control group at all follow-up assessments. The highest total numbers of non-smokers occurred in both study groups at the 12 months follow-up. At the 10 years follow-up, in observed cases-only analysis, the total number of non-smokers (the primary outcome of the Laocoon study) was 61/119 (51.3%) in the intervention group and 51/112 (45.5%) in the control group, p=0.38. The corresponding all-cases analysis with penalized imputation (non-responder classified as current smoker) yielded 61/505 (12.1%) non-smokers in the intervention group and 51/506 (10.1%) non-smokers in the control group, p=0.31.

Table 1 shows the results after re-analyzing the continual abstinence (secondary study outcome of the Laocoon study). Through the 12 months follow-up (adjusted Model 1), the intervention showed an independent statistically non-significant effect (OR=1.28, 95% CI 0.95–1.74; p = 0.103) but through the 10 years follow-up (adjusted model 2), the intervention showed a statistically significant effect (OR=1.32, 95% CI 1.01–1.73, p=0.042) ( Table 1). In this final model, other significant and independent associations of continual abstinence up to 10 years follow-up with baseline parameter were time to follow-up assessment (p<0.001 in all four models), smoking-related factors such as higher points on a motivation ladder and higher perceived self-efficacy to stop smoking, as well as less nicotine dependence as measured with the Fagerström test. Patients in the internal medicine subunit of the ED had higher odds of becoming non-smokers compared to surgical patients from the surgical subunit (p=0.027 up to 12 months follow-up and p=0.044 up to 10 years follow-up, respectively). The covariance parameter estimates for this final model showed nearly identical variance for the five measures in each of the four models, respectively, and declining correlations between consecutive assessments (Supplementary Table 1).

Table 2 compares baseline characteristics of study participants in the 10 years follow-up (follow-up responder) with those of follow-up non-responders who did not respond to any of three contact attempts. Table 2 additionally provides information on the baseline characteristics of study participants with no current addresses identified. Neither exposure to the smoking intervention at baseline, nor the smoking status up to 6 months after the intervention is associated with 10-year loss to follow-up (all p>0.05). Higher abstinence rates at 12 months follow-up (23.6% vs 15.9%, p=0.031) as well as older age at baseline (p<0.001), higher educational attainment (p=0.013) and smoking fewer cigarettes per day (p=0.013) at baseline and being less tobacco dependent (p=0.019) at baseline were significantly associated with study participation at the 10 years follow-up.

Table 3 shows the baseline characteristics of the 231 Laocoon-study participants, stratified by smoking status at the 10 years follow-up. While there was no difference in randomization status (p=0.38), those study participants who were tobacco-abstinent at the 10 years follow-up were smoking on average fewer cigarettes at baseline, were less nicotine dependent, showed higher motivation to quit smoking and reported lower frequency of harmful alcohol consumption (all p<0.05). Retrospective postdiction of abstinence status up to 12 months, for respondents not smoking at the 10 years follow-up rose from 16.3% at the 1 month follow-up to 36.8% at the 12 months follow-up while the corresponding abstinence for respondents who reported smoking at the 10 years follow-up rose from 3.2% at 1 month follow-up to 11.5% at the 12 months follow-up (differences between groups: all p<0.05). With regard to baseline social influences, tobacco abstainers at the 10 years follow-up compared with self-identified smokers reported more often having a non-smoking partner (42.9% vs 21.0%, p= 0.001) and having attained more years of education (p=0.035).

**Table 3:** Comparing 10-year non-smokers and current smokers in the Laocoon study on TED-study baseline characteristics (N=231)

*Baseline and follow-up measures (TED study)*	*10-year non-smoker N=112 (48.5%)*	*10-year smoker N=119 (51.5%)*	*p*
Intervention group, n (%)	61 (54.5)	58 (48.7)	0.38
Surgical subunit of the ED, n (%)	66 (55.5)	59 (52.7)	0.67
Non-smoker at 1 month follow-up (N=179) n (%)	14 (16.3)	3 (3.2)	0.003
Non-smoker at 3 months follow-up (N=179) n (%)	20 (25.0)	5 (5.1)	< 0.001
Non-smoker at 6 months follow-up (N=185) n (%)	23 (25.8)	12 (12.5)	0.021
Non-smoker at 12 months follow-up (N=199) n (%)	35 (36.8)	12 (11.5)	< 0.001
Age (years) mean ± SD, range	35.5 ± 12.2, (18–65)	34.3 ± 11.8, (18–68)	0.46
Female gender, n (%)	51 (45.5)	52 (43.7)	0.78
Number of cigarettes smoked per day mean ± SD, range	13.4 ± 9.5, (1–40)	16.5 ± 8.9, (1–35)	0.009
Fagerström test mean ± SD	2.5 ± 2.5	3.4 ± 2.6	0.008
Motivation to stop smoking 10-point scale, mean ± SD	5.9 ± 2.8	5.0 ± 2.6	0.058
**Motivation to stop smoking^a^, n (%)**			
unmotivated	59 (52.7)	76 (63.9)	
ambivalent	34 (30.4)	32 (26.9)	0.044^c^
motivated	19 (17.0)^b^	11 (9.2)	
Perceived self-efficacy to stop smoking 10-point scale, mean ± SD	5.5 ± 2.6	5.4 ± 2.9	0.16
Harmful alcohol consumption^d^, n (%)	31 (27.7)	50 (42.0)	0.022
Illicit drug use^e^, n (%)	57 (50.9)	70 (58.8)	0.23
**Partnership, n (%)**			
No partner	31 (27.7)	40 (33.6)	0.001
Smoking partner	33 (29.5)	54 (45.4)	
Non-smoking partner	48 (42.9)	25 (21.0)	
Living in a single household, n (%)	38 (33.9)	49 (41.2)	0.26
University-entrance diploma, n (%)	70 (62.5)	58 (48.7)	0.035
Net family income/month above median^f^ in 2006 (N=187) n (%)	31 (33.3)	41 (43.6)	0.15
Family doctor, n (%)	90 (80.4)	93 (78.2)	0.68

a ‘When do you wish to stop smoking?’ (‘Not within the next 6 months’=unmotivated smokers, ‘Within the next 6 months but not within the next 4 weeks’=ambivalent smokers and ‘Within the next 4 weeks’=motivated smokers)^10,11^.

b Does not sum up to 100% because of rounding error.

c Chi-squared test for trend.

d ≥5 points in the AUDIT-PC^12^.

e At least one single use within the last 12 months before the TED-study baseline: cannabis, ecstasy, other designer drugs, natural drugs (e.g. Peyote), cocaine, morphine, heroin or other opiates^8^.

f 1475€/month8. TED: Tobacco Control in an Urban Emergency Department^8^. SD: standard deviation. ED: emergency department. AUDIT-PC: Alcohol Use Disorders Identification Test–Piccinelli Consumption.

## DISCUSSION

The long-term follow-up of a randomized controlled trial involving emergency department-initiated tobacco control allowed analyzing both isolated as well as repeated measures of point-prevalence abstinence up to 10 years after the initial intervention. While there was no effect on the single point-prevalence outcome at the 10 years follow-up, evidence obtained from the more integrative modeling approach suggests a small cumulative impact on repeated point-prevalence abstinence across five time-points over the entire course of both studies. Moreover, several smoking-related baseline parameters were significantly associated with continual tobacco abstinence over 10 years follow-up. They included baseline level of nicotine dependence, motivation to quit smoking as well as perceived self-efficacy to quit.

This study is to our knowledge the first attempt to evaluate the impact of ED-initiated tobacco control on 10-year smoking status and to use continual tobacco abstinence as the outcome. Data did not exist on loss to follow-up or effect size for a 10-year follow-up before the Laocoon study. As far as we know, the longest duration of follow-up in previous RCTs involving ED-initiated smoking cessation was 12 months^4^. Considering the null results at the 12 months follow-up of the initial study, the single observation, longer-term result presented here may not be surprising. However, evidence from other medical settings suggests mixed effects of individually-focused tobacco control interventions on long-term tobacco abstinence. In a Chinese smoking cessation clinic abstinence rates rose from 27% at the 1 year follow-up to 38% at the 7 years follow-up^21^. Up to 8 years after termination of a 12-month RCT in a dental setting, cessation rates rose by 8 per cent and, although not statistically significant, differences between study arms persisted (p=0.16)^22^. Negative findings occurred in >2500 high school smokers seven years post-intervention, when motivational interviewing plus telephone counseling showed no impact on quit rates compared to usual care (14.2% vs 13.1%, p=0.61; respectively)^23^. Likewise, non-superiority occurred in an RCT with >1600 study participants that were prescribed the nicotine patch for smoking cessation compared with usual care eight years after study participation (non-smoking rates of 27.6% compared with 26.4%)^24^.

Individuals who smoke and present to emergency departments are typically more than a decade younger than patients who smoke and present in the clinical setting, with the latter often already suffering from tobacco-associated disease. Long waiting lines, the ED teachable moment, and the proven efficacy of brief smoking cessation intervention in the ED setting for up to one year are compelling arguments for implementing smoking cessation services into clinical routine in EDs^3^. The median counseling time in the ED of the TED study was 13 minutes and the median overall initial intervention, together with the four booster phone calls, did not exceed 30 minutes^8^. Thus, overall half an hour of individual face-to-face tobacco counseling in the ED in combination with booster phone calls seems to motivate this harder-to-reach smoking group to at least quit smoking temporarily. Because of the large number of young patients treated in EDs every day, implementation of brief smoking cessation interventions in this setting are likely to have a significant public health impact^3^. On an individual level and independently of age, gender, and educational status, baseline motivation and perceived self-efficacy to quit smoking as well as level of nicotine dependence were prognostic factors of continual tobacco abstinence in the results presented here. These smoking-related factors may be routinely evaluated using paper-based or electronic devices in those ED patients who are eligible for screening and brief intervention and who are not directly transferred to their medical treatment. Information about both baseline parameters helps in tailoring individual smoking cessation, e.g. according to the Clinical Practice Guidelines for Treating Tobacco Use and Dependence^25^.

An additional finding of note is the striking variability not only in smoking status, for those participants who chose to respond to each survey, but also in participants’ responsiveness to each survey. Switching between smoking, non-smoking and study-attrition/study-participation appears to be the norm and continual abstinence (or continual smoking) the exception. Early cessation is no guarantee of long-term abstinence but early relapse is also no guarantee of long-term smoking. It now seems premature to assume that no further follow-up outcome data will be available from a participant who failed to participate in an earlier follow-up assessment. Thus, the idea of capturing one single well-defined and validated study outcome as a proxy for overall success/failure of a tobacco control intervention seems overly pessimistic. Episodes of smoking abstinence increased significantly over time even among smokers in the control group. The significant independent effect of time may reflect the cumulative impact of periodic public health tobacco control campaigns and repeated exposure to other brief clinical interventions. It may also reflect the maturing out of smoking behavior from young adulthood into middle age, independently of a person’s individual life course and his/her interaction with the health care system, as well as specific time-period related factors, such as the new availability of e-cigarettes.

### Limitations

Although attrition status was not differentially associated with randomization status, the Laocoon-study follow-up participants were documentably not representative of the original study sample. Overall, compared to non-participants, participants in the Laocoon study were more often characterized by ‘protective’ characteristics such as older age (younger age was shown to impede the impact of individual tobacco counseling in different settings)^26-29^, lower nicotine dependence/fewer cigarettes smoked (a strong predictor for cessation success in various settings)^26,30-32^, more years of attained education (a known predictor of both success after smoking cessation^33^ as well as higher study adherence in various settings including tobacco control studies)^26,34-36^. In univariable comparisons (Table 3), this bias was not taken into account and thus, the results have to be interpreted with caution. In the multivariable models however (Table 1), attrition as a potential source of bias was addressed by adjusting for parameters associated with non-response in univariate analysis. Nevertheless, the results presented here have to be regarded as not only deriving from a secondary data analysis, but as being the product of exploratory analyses. This is why p-values were not adjusted for multiple testing.

The high rate of loss to follow-up between the 12 months and the 10 years follow-up was to be expected for such a long interval, despite several attempts, including regulatory inquiries, to increase the number of correct addresses and thus the number of potential follow-up participants. A response rate of 23% (in regard to persons with current postal address) is notwithstanding a significant weakness of this study, that may not be satisfactory eliminated with elaborate analytical approaches.

More confident inferences about modifiable influences on the impact of smoking cessation in the emergency department setting over time await studies that minimize attrition through implementation of more rigorous follow-up protocols. There is furthermore a need for biological validation of smoking status outcomes. While smoking outcomes were biologically validated at the 12 months follow-up of the parent study^8^, this was not feasible during this postal long-term follow-up.

## CONCLUSIONS

Future research in this setting should consider the course of ED patients’ compliance/non-compliance evaluated in this specific study setting. The unstable findings involving smoking/non-smoking status highlights the need for analytical approaches that take into account this dynamism of outcome. Research would probably benefit from repeated assessments of 7-day point prevalence smoking status, capturing more adequately the picture of the natural course of the smoking behavior over time. Furthermore, future research should evaluate if the benefit of ED-initiated tobacco control increases with more specifically tailored interventions and more rigorous treatment of tobacco dependence.
