# Supplementary material for: Secondary analysis of an RCT on Emergency Department-Initiated Tobacco Control: Repeatedly assessed point-prevalence abstinence up to 12 months and extension of results through a 10-year follow-up
Source: Tob Induc Dis. 2019 Apr 5;17:26. doi: 10.18332/tid/105579 (PMC6751984; doi:10.18332/tid/105579)
Supplement: Supplementary file 1 [file TID-17-26-s1.pdf]

Supplement Figure 1. Total number of non-smokers over all follow-up assessments for the TED and Laocoon studies (N=1011)

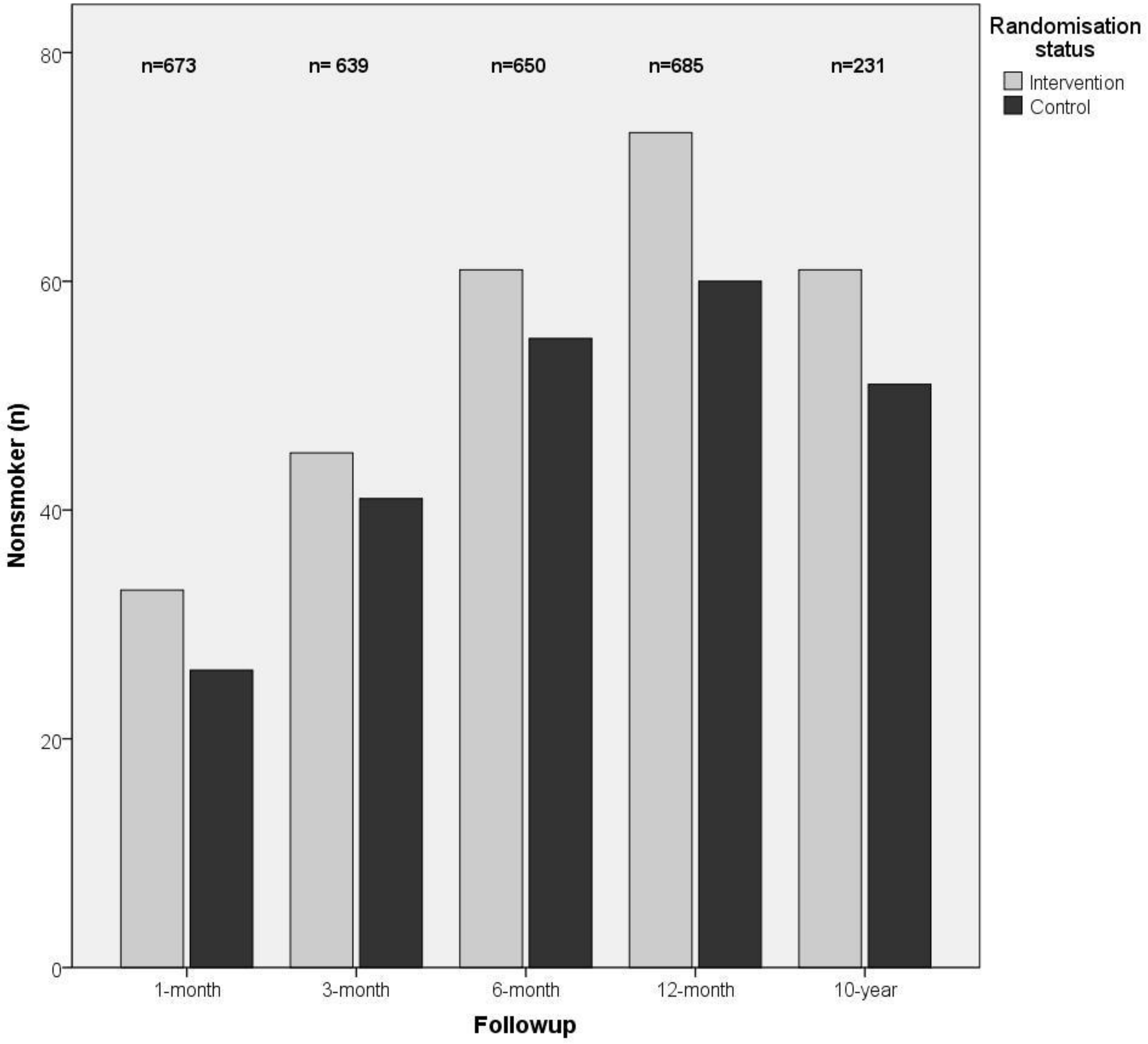

© 2019 Weiss-Gerlach E.
