# Supplementary material for: Secondary analysis of an RCT on Emergency Department-Initiated Tobacco Control: Repeatedly assessed point-prevalence abstinence up to 12 months and extension of results through a 10-year follow-up
Source: Tob Induc Dis. 2019 Apr 5;17:26. doi: 10.18332/tid/105579 (PMC6751984; doi:10.18332/tid/105579)
Supplement: Supplementary file 2 [file TID-17-26-s2.pdf]

Supplement Table 1.

Covariance parameter estimates for the adjusted GLMMs of repeated point prevalence abstinence through 12 months and through 10 years, respectively (N=1011)

	Follow-up through 12 months only		Follow-up through 10 years	
Measurement at	Variance σ^2	Correlation parameter rho (ρ)	Variance σ^2	Correlation parameter rho (ρ)
1 month follow-up	1.14	1 month/3 months: 0.56	1.08	1 month/3 months: 0.56
3 months follow-up	0.97	3 months/6 months: 0.39	0.94	3 months/6 months: 0.39
6 months follow-up	1.09	6 months/12 months: 0.28	1.02	6 months/12 months: 0.30
12 months follow-up	1.04	-	1.22	12 months/10 years: 0.28
10 years follow-up	-	-	0.99	-

© 2019 Weiss-Gerlach E.
